# The ongoing need for NHS gambling harms services in Wales: time to put words into action

**DOI:** 10.3389/fpsyt.2023.1277435

**Published:** 2023-10-25

**Authors:** Simon Dymond, Glen Dighton, Alice E. Hoon, Gareth Roderique-Davies, Bev John, Henrietta Bowden-Jones

**Affiliations:** ^1^School of Psychology, Swansea University, Swansea, United Kingdom; ^2^Department of Psychology, Reykjavík University, Reykjavík, Iceland; ^3^Swansea University Medical School, Swansea, United Kingdom; ^4^Faculty of Life Sciences and Education, University of South Wales, Treforest, United Kingdom; ^5^National Problem Gambling Clinic and National Centre for Gaming Disorders, London, United Kingdom; ^6^Department of Psychiatry, University of Cambridge; Cambridgeshire and Peterborough NHS Foundation Trust, London, United Kingdom

**Keywords:** gambling, clinic, Wales (UK), treatment, harm

With referrals to the NHS Gambling Harms Services increasing quarter on quarter, year on year, to 1,400 in 2022, a further seven gambling harms clinics have now opened in England bringing the total number to 15 (1). Yet, the number of clinics open in Wales and Scotland remains zero. Here, we argue that the ongoing absence of NHS clinics in Wales is unacceptable and must be urgently addressed by the Welsh Government as part of its devolved health and social care portfolio.

People in Wales are just as much at risk of experiencing harms from gambling as anywhere else. In 2018, the Welsh Problem Gambling Survey identified that 52% had gambled in the past year, and 3.6% or approximately 113,000 people experienced some harm from their gambling (2). This estimate omits the even higher numbers of affected others impacted by gambling-related harm. Data from the National Survey for Wales 2022 indicated that gambling participation has increased since 2018, with 63% having gambled within the last 12 months (3). Moreover, 4% of respondents felt that they had both bet more than they could afford and had gone back to try to win money that they had lost (i.e., “loss-chasing”); these behaviors are indicative of clinically significant levels of harmful gambling. Taken together, these prevalence estimates, representing 120,000 people, highlight increased numbers of the Welsh population either experiencing or at risk-of gambling-related harm. With the widespread availability of opportunities to gamble on increasingly riskier and heavily advertised products, this is a trend that looks set to continue.

Gambling-related harm has long been acknowledged as a public health concern for Wales. Following calls from the Chief Medical Officer for Wales (4) and key stakeholders (5), in 2022 Public Health Wales commissioned a Health Needs Assessment (HNA) focusing on gambling (6). The HNA highlighted challenges surrounding the awareness, accessibility, and acceptability of current gambling treatment and support provision in Wales. It found that many individuals and affected others grappling with gambling-related harms were not aware of the support available from charities and third-sector providers and that they faced barriers in accessing these services or hesitated seeking help due to perceived shame and stigma. Overcoming these challenges to help-seeking may be further compounded by the rurality of Wales. Indeed, the geospatial distribution of those experiencing gambling-related harms and those seeking support includes both primarily rural areas such as Rhondda Cynon Taf, and metropolitan conurbations around cities such as Bangor, Wrexham, and Cardiff (5, 7). In Wales, as elsewhere in England (8), gambling-related harm is therefore not an experience unique to urban areas. Accessing help and support, however, may depend on where one lives and how connected one is to larger, more urban areas.

At present, the provision of treatment and support for gambling-related harms in Wales relies exclusively on third-sector organizations. Gamblers' Anonymous meetings are held in several locations across Wales. Adferiad Recovery has offices in Swansea and in-patient treatment facilities in Colwyn Bay, while ARA Recovery, which is primarily based along the M4 corridor spanning Wales and England, currently has drop-in services in Cardiff, Maesteg, Swansea, and Carmarthen. Both charities are part of the National Gambling Support Network, administered by GamCare, which is funded by GambleAware. According to GambleAware's Annual GB Treatment and Support Survey 2022, which included participants from Wales, only 16% of respondents that experience any level of gambling-related harm reported using any type of formal treatment (9). One key barrier to accessing support or treatment again involved perceived stigma of harmful gambling, whilst knowing how to access support, including being able to self-refer or access the support through a specific channel (e.g., face-to-face) was one of the biggest motivators of reaching out (9). Previous reports from GambleAware provided detailed analysis of region-specific treatment needs and highlighted that the 4% referral rate to the Network from Wales (10) is a stable but gradually increasing trend (see Figure 1). Importantly, however, these referral figures are the tip of the iceberg and do not reflect the estimated 120,000 Welsh people (2, 3) who do not come forward for health and support, or indeed the vast swathes of affected others impacted by someone else's harmful gambling.

**Figure 1:**
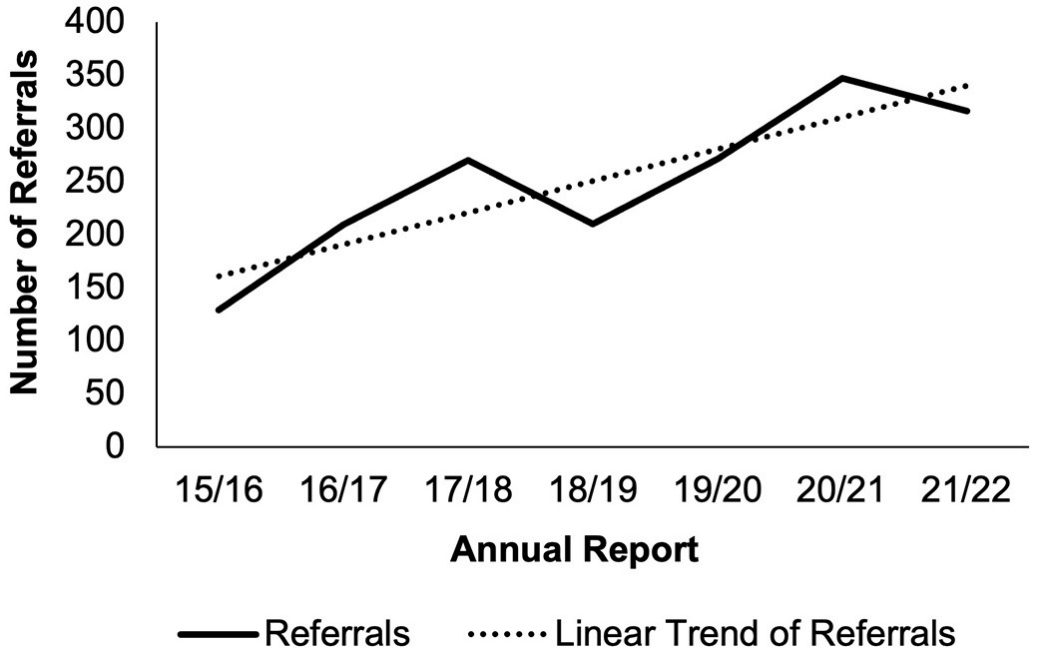
Persons living in Wales referred to the national gambling treatment service (10, 12).

Recent draft guidelines issued by the UK's National Institute for Health and Care Excellence (NICE) on the identification, assessment and treatment of people who may be harmed by gambling (11), recommends that people with high gambling severity scores (i.e., a score of 8 or more on the Problem Gambling Severity Index) should seek treatment and support from NHS-commissioned specialist gambling treatment services. Similarly, NICE guidelines recommend that people with lower gambling severity scores and complex harms and/or psychiatric comorbidities should also be referred to specialist NHS-commissioned gambling treatment services. Providing NHS-based services in Wales, free at the point of access, that are private and confidential, may also help overcome stigma and encourage people experiencing gambling harm to continue with treatment (9). Indeed, having gambling treatment services available in separate locations may encourage wider access. Moreover, and importantly given the ongoing cost of living crisis, the resource and cost implications of setting up these new services are likely to be offset by the cost savings to the NHS and society more broadly by effectively treating harmful gambling within any proposed new NHS gambling treatment services in Wales. Overall, then, the NICE-endorsed provision of NHS gambling services caters for service users with and without complex needs and comorbidities, fosters wider engagement with stigmatized or hard-to-reach groups, and will likely deliver considerable cost savings.

In Wales, we find ourselves in the situation where the Public Health Wales HNA report acknowledged a role for NHS specialist gambling treatment services, where the UK Government's Gambling White Paper stated that the outcomes of the HNA should “inform the development of specialist treatment services in Wales” (p. 132) (13), and where the Welsh Government's own Task and Finish Group on Gambling-Related Harms recommended that it “develop a clear referral pathway and… deliver a specialist gambling treatment service for Wales (14).” And yet, the wait for specialized gambling treatment, help and support services in Wales continues (15), while there is clearly both the need and expectation from the population for more and that Wales could do better. Political and public health intention now, more than ever, needs to be mobilized into delivery.

It is worth noting that establishing NHS gambling harms clinics in England was initially achieved with a co-commissioned service delivery model. Such an approach remains an important means of achieving the long-term goals of the NHS as they relate to gambling treatment and support (1). As we in Wales and elsewhere in GB transition from a voluntary levy to a statutory levy on gambling industry profits, organizations capable of delivering gambling harm prevention, support, and treatment services must be adept at securing funding for frontline services in new and sustainable ways.

In conclusion, we maintain that Wales should have also have equal and assured access to all possible service delivery models if it is to address the current glaring gap in provision. The need is acute, and we call on the Welsh Government and key stakeholders to put words into action and give the Welsh people a specialized gambling harms treatment service that meets their needs.

## Author contributions

SD: Conceptualization, Funding acquisition, Supervision, Writing—review and editing. GD: Writing—original draft, Writing—review and editing. AH: Funding acquisition, Writing—review and editing. GR-D: Funding acquisition, Writing—review and editing. BJ: Funding acquisition, Writing—review and editing. HB-J: Funding acquisition, Writing—review and editing.
